# Micropatterning of Confined Surfaces with Polymer Brushes by Two‐Photon‐Initiated Reversible Addition–Fragmentation Chain‐Transfer Polymerization

**DOI:** 10.1002/smsc.202400263

**Published:** 2024-11-21

**Authors:** Stefan Helfert, Tommaso Zandrini, Andreas Rohatschek, Manuel Rufin, Peter Machata, Anna Zahoranová, Orestis G. Andriotis, Philipp J. Thurner, Aleksandr Ovsianikov, Robert Liska, Stefan Baudis

**Affiliations:** ^1^ Institute of Applied Synthetic Chemistry TU Wien Getreidemarkt 9/163MC 1060 Vienna Austria; ^2^ Austrian Cluster for Tissue Regeneration 1060 Vienna Austria; ^3^ Institute of Materials Science and Technology TU Wien Getreidemarkt 9/308 1060 Vienna Austria; ^4^ Christian Doppler Laboratory for Advanced Polymers for Biomaterials and 3D Printing TU Wien 1060 Vienna Austria; ^5^ Institute of Lightweight Design and Structural Biomechanics TU Wien 1060 Vienna Austria; ^6^ Department of Composite Materials Polymer Institute of the Slovak Academy of Sciences Dúbravská cesta 9 84541 Bratislava Slovakia

**Keywords:** atomic force microscopy, micropatternings, reversible deactivation radical polymerizations, surface modifications, two‐photon polymerizations

## Abstract

Photopatterned polymer brushes provide a viable option to alter the surface properties of biosensors, substrates for tissue engineering, or microelectronic implants. Although the one‐photon direct laser writing enables excellent control over pattern geometry, it has an inherently limited writing resolution caused by the used light source; moreover, no patterning of undercuts or channels is possible. This article describes the preparation of patterned polymer brushes on confined glass substrates using two‐photon‐initiated reversible addition–fragmentation chain‐transfer (2PRAFT) polymerization of *N*‐acryloylmorpholine as a hydrophilic model monomer. The polymer brushes prepared by 2PRAFT exhibit a height of 10 nm, as confirmed by atomic force microscopy. In addition, well‐defined printed structures down to 5 μm size are prepared, which outperforms the currently achieved resolution of polymer brushes prepared by one‐photon direct laser writing.

## Graphical abstract

Two‐photon‐initiated reversible addition–fragmentation chain‐transfer (2PRAFT) polymerization enables the modification of surfaces with polymer brushes with defined micropattern and chemistry. Compared to other preceding techniques, like digital light processing‐based photoinduced electron transfer–RAFT or direct UV laser writing, 2PRAFT is capable of the modification of confined surfaces, for example, microfluidic channels with different chemistries and micropattern, respectively.

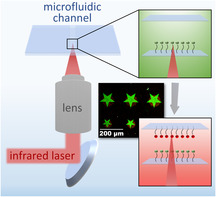

## 1 Introduction

Using a (bio)material in contact with a biological environment requires precise control over the materials’ surface properties to guarantee its desired biocompatibility and functionality.^[^ ^1^ ^]^ A feasible way to alter the surface properties is to functionalize it with polymer brushes. In this way, antifouling surfaces to avoid nonspecific protein and cell adhesion or, oppositely, bioactive materials, which interact with biological systems in a predictable and controlled manner, can be achieved. The polymer brushes are attached to the surface by “grafting to” approach, where the prepolymerized chains are attached to the surface, or “grafting from” methods, where the polymer chains are grown directly from functionalized surfaces. Surface‐initiated “grafting from” methods often include reversible deactivation radical polymerization (RDRP) techniques since these techniques provide brushes with high grafting densities and well‐controlled uniform brush thickness.^[^ ^2^ ^]^ Among the various RDRP techniques, atom‐transfer radical polymerization^[^ ^3^, ^4^ ^]^ and reversible addition–fragmentation chain‐transfer (RAFT) polymerization^[^ ^5^, ^6^ ^]^ are the most widely used for the preparation of surface‐grafted polymer brushes.

Specific applications, such as biosensors, substrates for cell cultivation, implants with microelectronic active parts, or microfluidic devices, require the preparation of (micro)patterned polymer brushes, for example, for spatial control over surface wettability. Micropatterned polymer brushes can be easily prepared by light using a photomask, externally projected light pattern, or direct laser writing.^[^ ^7^ ^]^ While photolithographic techniques represent the most frequently used and relatively convenient method of micropatterning the surfaces, the selected photomask limits the final shape and geometry of the pattern. More importantly, the photolithographic techniques are only applicable to relatively flat and open substrates. Surfaces with exceeding roughness and/or undercuts are not applicable. As an alternative, Laun et al.^[^ ^8^ ^]^ employed direct laser writing to gain more freedom in the design of polymer brushes. However, the achieved resolution was only 270 μm due to the used type of laser.

**Figure 1:**
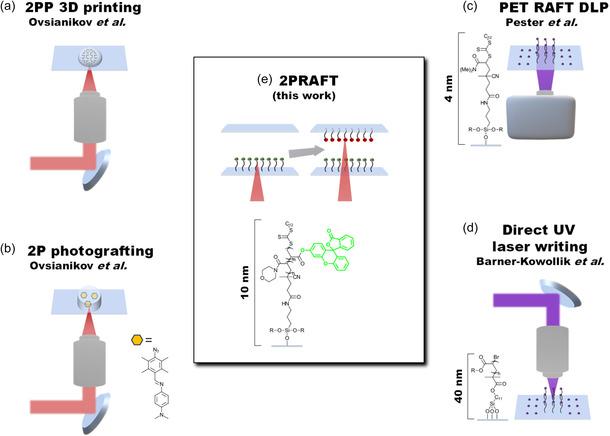
Overview of the previously published methods in comparison to the current work. a) During 2PP, a liquid formulation is solidified by crosslinking.^[^ ^10^, ^11^, ^12^ ^]^ b) 2P grafting involves a previously generated gel, where a grafting agent was swelled inside to 3D write a chemical trace into the material.^[^ ^14^, ^15^ ^]^ Photosurface grafting was performed by immobilization of c) RAFT agents^[^ ^16^ ^]^ or d) ATRP initiators.^[^ ^8^ ^]^ e) In this work, we present 2PRAFT with the possibility to modify different surfaces of the same device with different chemistries.

This work addresses the inherently lower resolution of one‐photon (UV) direct laser writing by employing two‐photon (2P) absorption to prepare patterned polymer brushes via 2P‐initiated RAFT (2PRAFT) polymerization. Since the 2P absorption process exhibits nonlinear intensity dependence, the excitation is localized within the focal volume of the laser beam, enabling higher spatial resolution of the patterning even inside structures.^[^ ^9^ ^]^ We would like to emphasize the difference of our proposed method in comparison to already published approaches (overview in **Figure** **1**). Part of our group has extensive experience with 2P lithography, where crosslinked 3D structures are printed.^[^ ^10^, ^11^, ^12^, ^13^ ^]^ Here, in contrast, we focus on preparing linear surface‐attached polymer brushes employing “grafting from” method. Ovsianikov et al.^[^ ^14^, ^15^ ^]^ published a method for the functionalization of surfaces by low‐molecular‐weight compounds using 2P photografting. In comparison to surface modification with low‐molecular‐weight compounds, the modification with polymer brushes allows better control over surface properties (e.g., hydrophobicity, protein adhesion) and can lead to a higher density of the active compounds on the surface. As discussed above, in comparison to direct one‐photon writing,^[^ ^8^ ^]^ our method provides superior resolution and allows printing on specific arrangements (e.g., channels). Pester, Boyer, and co‐workers developed surface‐initiated photoinduced electron transfer‐reversible addition−fragmentation chain‐transfer (SI‐PET‐RAFT) polymerization method to create patterned surfaces using photomask^[^ ^5^ ^]^ and, more recently, digital projection patterning.^[^ ^16^ ^]^ However, both methods have limited use in the case of selective patterning in confined spaces, such as in microfluidic devices.

In the present work, we selected hydrophilic and biocompatible poly(*N*‐acryloylmorpholine) (pNAM) to produce polymer brushes. This polymer was previously used to prepare patterned surfaces for controlled cell attachment using a photomask.^[^ ^17^ ^]^ First, we studied the kinetics of the formation of polymer brushes (without patterning) by RAFT polymerization using blue‐light irradiation, to optimize the composition of the polymerization mixture. The brush thickness was measured using ellipsometry and the chemical composition was determined using X‐ray photoelectron spectroscopy (XPS). After initial irradiation experiments, we moved towards 2PRAFT surface patterning. We analyzed the formed brush patterns on the surface using a confocal laser scanning microscope (CLSM) and atomic force microscopy (AFM). To further highlight the possibilities of 2PRAFT, printing of patterns of different colors and printing on vertically stacked surfaces were performed.

## 2 Results and Discussion

Although the ultimate goal of the presented work was to establish 2P‐initiated RAFT polymer brush formation, it was necessary to perform preliminary optimizing experiments using blue light‐induced RAFT photopolymerization. The reason for this decision is the availability of fast and facile methods for brush thickness measurements (ellipsometry) and the possibility to characterize the chemical composition of the surface using XPS. These methods are not applicable to the characterization of 2P patterned surface as the printing of shapes with the required size using IR laser is not feasible.

### 2.1 One‐Photon RAFT Polymerization of pNAM Brushes

**Figure 2:**
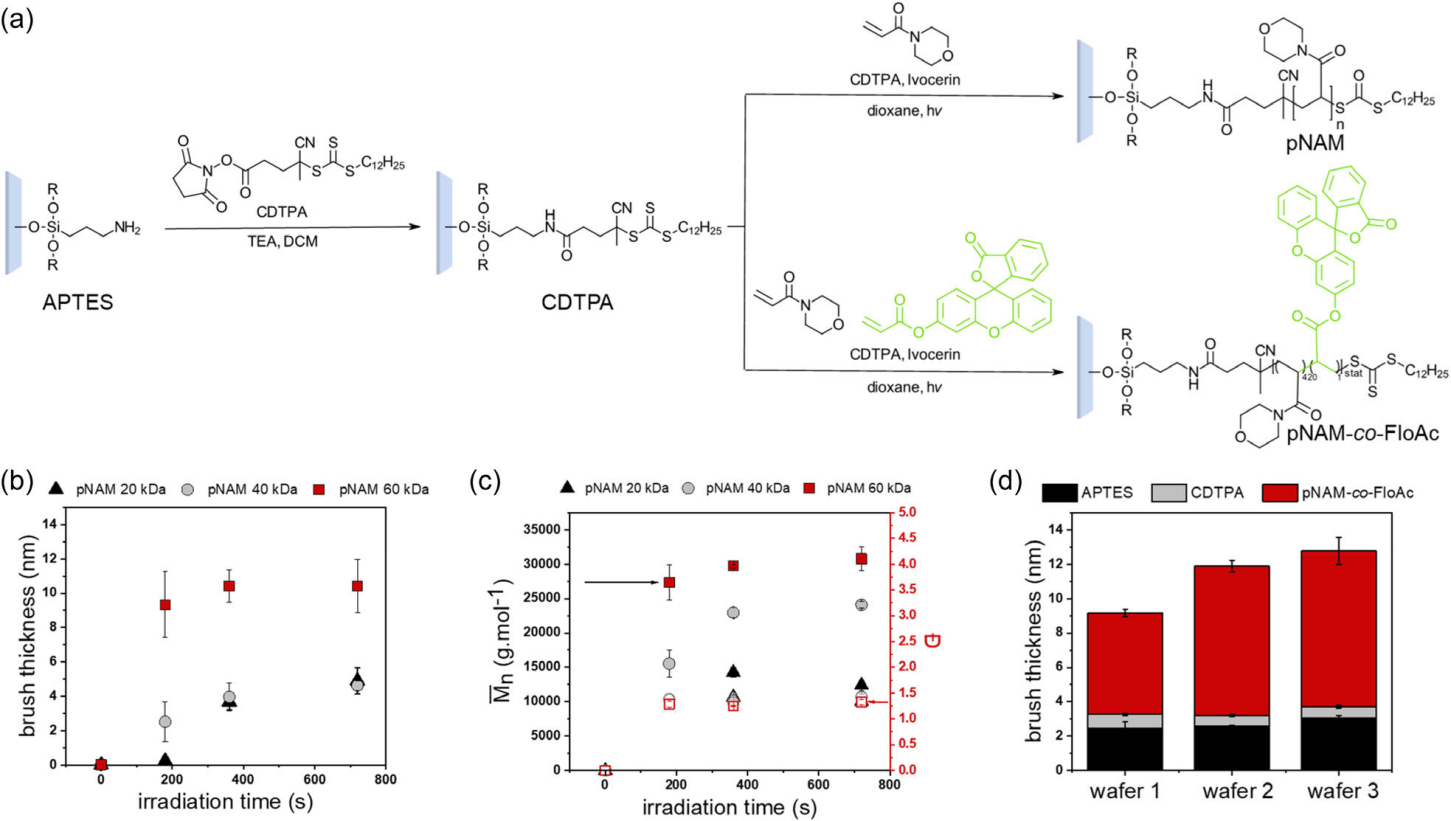
a) Schematic representation of photopolymerization of NAM brushes, grafted from Si wafers covalently modified with RAFT agent CDTPA, b) the dependence of the dry brush thickness measured by ellipsometry on irradiation time (wavelength 400–500 nm) for 3 different targeted $\bar{M_{n}}$ 20, 40, and 60 kDa, and c) the dependence of the number average molar mass $\bar{M_{n}}$ (filled symbols) and dispersity *Ð* (open symbols) of free polymers collected from the wafer surface of 3 different targeted $\bar{M_{n}}$ 20, 40, and 60 kDa. b,c) The values are displayed as mean ± SD from 4–5 individual wafers. d) Three‐step preparation of 60 kDa pNAM‐*co*‐FloAc brushes: dry thickness of each step measured by the ellipsometry; the values are displayed as mean ± SD from 6 spots on the wafer.

We started with the preparation of blue‐light photopolymerized pNAM brushes from model Si wafers to optimize the formulation composition. Prior to the photopolymerization, Si wafers were covalently modified with RAFT agent 4‐cyano‐4‐(((dodecylthio)carbonothioyl)thio)pentanoic acid (CDTPA, **Figure** **2**a) by a two‐step procedure. An efficient visible light photoinitiator Ivocerin^[^ ^18^ ^]^ (bis‐(4‐methoxybenzoyl)diethylgermanium, PI) was selected for these photopolymerization optimization experiments as a light source with the wavelength in the range from 400 to 500 nm and an intensity of 28.8 mW cm^−2^ was used. The formulations further contained NAM monomer (4 M concentration in dioxane) and free CDTPA with three different molar ratios of NAM:CDTPA:PI (140:1:0.25, 280:1:0.25, 420:1:0.25). The ratio of NAM:CDTPA also determines the targeted number average molar mass $\bar{M_{n}}$ of resulting pNAM polymers (in this case, 20, 40, and 60 kDa, respectively). The dry thickness of prepared brushes was examined by ellipsometry (Figure 2b).

After irradiation for 180 s, brush thicknesses reached almost the maximum values for all three formulations, indicating fast polymerization. The maximum brush thickness of 10.4 ± 1.5 nm was achieved for the ratio NAM:CTA 420:1 (targeted $\bar{M_{n}}$ 60 kDa); this formulation was thus selected for further experiments. In addition to dry brush thickness, we also measured $\bar{M_{n}}$ and the dispersities *Ð* of free polymer collected from the solution above the wafers (Figure 2c). Although these free polymers have a systematically different molecular weight compared to the surface tethered brushes, they enable a very easy qualitative assessment of the RAFT polymerization. In general, $\bar{M_{n}}$ increased with increasing NAM:CDTPA ratio. This trend was more pronounced than in the case of dry brush thickness, where the differences between 20 and 40 kDa formulations were negligible. In the case of the shortest irradiation time of 180 s of the 20 kDa formulation, only very low $\bar{M_{n}}$ values were measured, indicating no polymerization under these conditions. This finding corroborates well with the dry brush thickness measurement. In general, the brush thickness increased steadily with the measured $\bar{M_{n}}$ values (Figure S1, Supporting Information), although they differ from the targeted values, which can be attributed to the lower maximum conversion of this RAFT system (Figure S2 and S3, Supporting Information) combined with the effect of conventional gel permeation chromatography (GPC) calibration on polystyrene standards (PSS). *Ð* values below 1.5 indicate good control over the polymerization.^[^ ^19^ ^]^ The obtained value for $\bar{M_{n}}$, the brush thickness, and the radius of gyration (obtained from dynamic light scattering experiments)^[^ ^20^ ^]^ were employed to calculate the reduced tethered density, which suggest that brushes in the highly stretched regime could be obtained (more details are available in the Section S2, Supporting Information).

The self‐limited surface thickness of polymer brushes was also reported in the works of Postnikov et al.^[^ ^21^, ^22^ ^]^ They prepared poly(*N*‐isopropyl acrylamide) layers on a golden surface via RAFT polymerization and observed a self‐limiting polymerization mechanism. The authors discussed that the reason was the coverage of the golden surface by the polymer layer, which blocks the generation of new radicals via the plasmon mechanism. However, we have seen a similar effect also for our reaction in solution (Figure S2, Supporting Information). Similarly, to the brush thickness dependence on irradiation time, also ln (*M*_0_/*M*_t_) increases linearly for the first 200 s, suggesting a controlled mechanism of polymerization. However, for the longer irradiation times, ln (*M*_0_/*M*_t_) reaches a plateau. Interestingly, a similar effect for the formation of poly(*N*‐acryloylmorpholine) brushes via RAFT polymerization was described by Mutlutürk et al.^[^ ^23^ ^]^ where the authors reported the formation of brushes with a maximum of 17 nm; with longer irradiation times, the brush thickness was plateauing.

To enable direct visualization of patterned pNAM brushes by fluorescence microscopy, we included very small amounts of fluorescent comonomer, fluorescein‐*o*‐acrylate (FloAc, Figure 2a). The used formulation consisted of NAM:CDTPA:FloAc:PI in a molar ratio of 420:1:1:0.25 (4 m NAM concentration in dioxane). The wafers were irradiated with a 400–500 nm light source with an intensity of 28.8 mW cm^−2^ for 360 s. On these samples, we focused on measuring the dry layer thickness by ellipsometry after each modification step (APTES modification, CDTPA‐NHS attachment, pNAM‐*co*‐FloAc brush formation). The results from three separate wafers are compared in Figure 2d, indicating good reproducibility. After APTES modification, the thickness increased to 2.6 ± 0.5 nm (wafer 2). An additional layer thickness of the CDTPA‐modified surface was 0.6 ± 0.06 nm. Finally, the pNAM‐*co*‐FloAc brush featured an additional thickness of 8.7 ± 0.4 nm. The achieved dry brush thicknesses correspond well with the previous experiments without fluorescent comonomer (10.4 ± 1.5 nm), indicating that the presence of comonomer does not drastically affect the RAFT polymerization.

**Figure 3:**
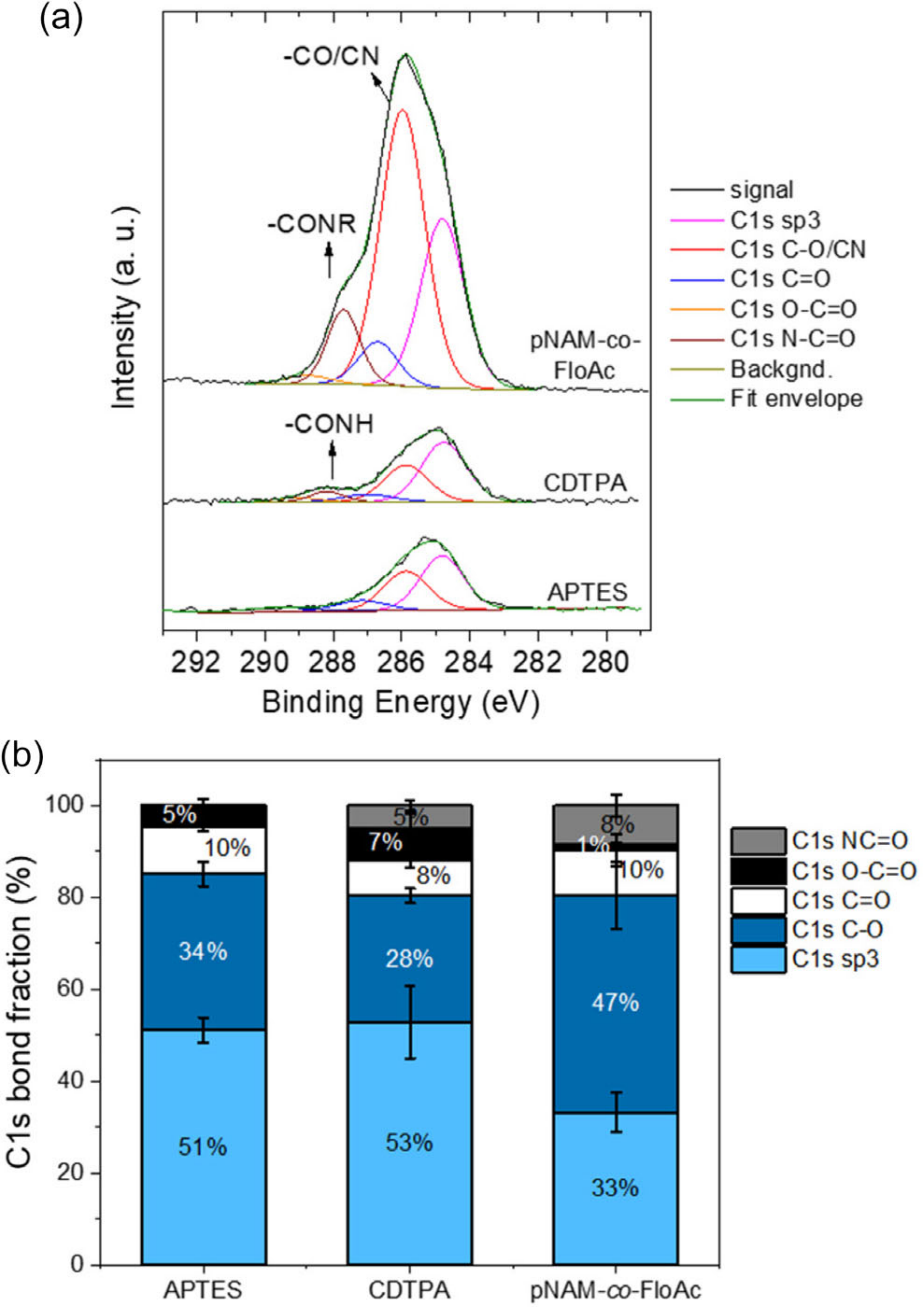
XPS analysis of wafers modified with APTES, CDTPA, and pNAM‐*co*‐FloAc a) C1s spectra after deconvolution and b) composition of carbon bonds in percentage. The values are displayed as mean ± SD from three different points on individual wafers. More details on the XPS measurements are available in Section S5 (Supporting Information).

To confirm the successful chemical modification of the model Si wafers, XPS measurement of all modification steps was performed (**Figure** **3**a). XPS survey spectra of APTES, CDTPA, and pNAM‐*co*‐FloAc 60 kDa are shown in Figure S7, Supporting Information. C1s bond fraction expressed as mean ± SD of 3 measurements at 3 different points is shown in Figure 3a (N1s and O1s spectra are shown in Figure S6, Supporting Information). The spectra after APTES modification show the major contribution of the sp3 carbon with a maximum at 284.8 eV (51.1 ± 2.8%) and the signal at 285.9 eV (34.0 ± 2.6%) as a result of C‐N bond between a carbon atom and an amino group. The resulting spectrum after CDTPA modification shows a significant increase in the XPS signal at 288.2 eV (from 0 to 4.9 ± 1.1%), confirming the presence of the amide group N─C=O in CDTPA. After photoRAFT polymerization, the resulting spectrum of the product indicates an increase in carbon content from 19.8 to 65.0% (Table S1, Supporting Information). The highest contribution to the signal in the C1s region after deconvolution (47.3 ± 7.3%) can be attributed to the presence of the C─O/C≡N group at 286.3 eV (Figure 3b). This peak is shifted compared to the spectra after modification with CDTPA and APTES, respectively, probably due to overlapping of C─O and C≡N signal, which cannot be easily distinguished. The higher contribution of the C1s signal at 288.0 eV (8.4 ± 2.4%) probably originates from the amide group as a part of NAM moiety (Figure 3b).

### 2.2 Two‐Photon RAFT Polymerization of pNAM Brushes

Encouraged by results from one‐photon RAFT, we moved forward to employ our optimized formulation for 2PRAFT. To achieve this goal, we had to change the photoinitiator Ivocerin to (2E,6E)‐2,6‐bis(4‐(dimethylamino)benzylidene)‐4‐methylcyclohexanone (M2CMK), an already used efficient photoinitiator suitable for 2P polymerization (2PP) fabrication,^[^ ^24^, ^25^ ^]^ due to the practical 2P inactivity of Ivocerin. This photoinitiator is widely used in 2PP‐related applications and its spectral properties as well as 2P absorption are reported elsewhere.^[^ ^24^, ^25^ ^]^ M2CMK exhibit suitably strong 2P absorption at 800 nm and broad processing window.^[^ ^24^ ^]^ This was the wavelength also used for our printing experiments.

**Figure 4:**
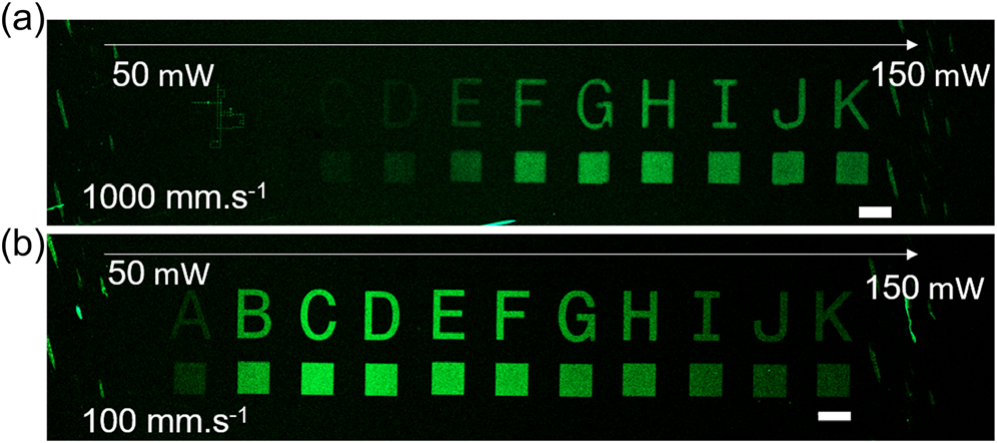
CLSM images of the 2PRAFT polymerization with the 4 m formulation of NAM and FloAc in dioxane at different writing speeds, a) 1000 mm s^−1^ and b) 100 mm s^−^^1^, and laser powers (A–K); the scale bar represents 100 μm.

Also, the model substrate was changed from Si wafers to microscope coverslips to enable laser exposure through glass, and later examination of the samples under an inverted CLSM. Except for the change of the photoinitiator, the formulation composition was kept similar as in previous experiments, that is, NAM:CDTPA:FloAc:M2CMK, 420:1:1:1 in dioxane (4 m). The printing pattern consisted of letters from A to K (100 μm thick); each letter was accompanied with a cube (100 × 100 × 100 μm). Initially, we focused on optimizing the printing parameters, specifically the writing speed and the laser power. **Figure** **4** shows the printing results for the two examined printing speeds 1000 and 100 mm s^−1^, with the laser power gradually increasing from 50 to 150 mW.

The printed structures are clearly visible under CLSM, proving the successful 2PRAFT of the polymer brushes. Higher fluorescence intensity of printed structures was achieved for the lower writing speed of 100 mm s^−1^, compared to 1000 mm s^−1^. Regarding the laser power, the highest fluorescence intensity, corresponding to the best grafting performance, was at 80 mW for the writing speed of 100 mm s^−1^ (letter D). This finding is unexpected, since the highest fluorescence intensity did not correlate with the highest laser power. This result could be explained by several effects. We hypothesize that thermal expansion effects within the formulation could decrease the concentrations of the reactants within the excited volume, impairing the RAFT polymerization. Also, the extensive heating of the polymerization mixture could lead to the thermal degradation of RAFT agent and formation of the “dead” polymer chain ends.^[^ ^26^, ^27^ ^]^ Finally, the higher light dose could lead to extensive formation of radicals, which can also lead to RAFT agent cleavage.^[^ ^28^ ^]^ Analogously, the thiol‐ene 2PP‐crosslinked systems containing gelatin‐norbornene showed a similar dependence of stiffness on laser power, where the highest indentation modulus was measured for medium laser powers.^[^ ^10^ ^]^ Overall, the identified optimum printing parameters for the formulation were the writing speed of 100 mm s^−1^ and laser power of 80 mW.

### 2.3 Multichemistry Micropatterning of Confined Surfaces by 2PRAFT

**Figure 5:**
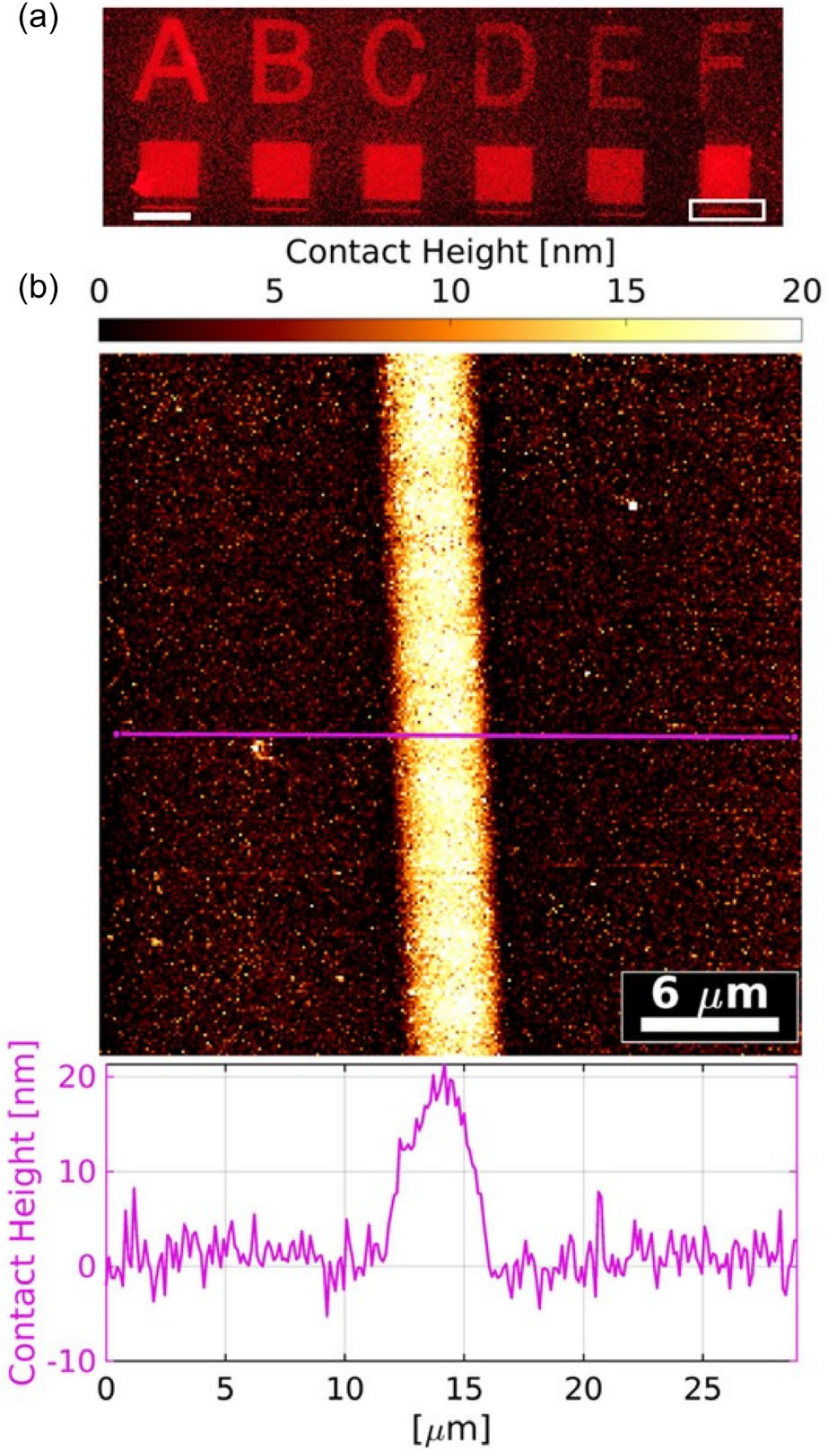
CLSM and AFM CP images of the 2PRAFT polymerization with the 4 m formulation containing NAM and NBAAm at scanning speed 100 mm s^−1^ and power 80 mW. a) The section highlighted by the white rectangle represents the AFM‐imaged area, scale bar 100 μm. 30 × 30 μm AFM CP image of 2PRAFT polymerized 5 μm wide line of polymer brushes imaged in PBS. Corresponding cross section of a selected region (purple line) is shown under the image (b).

We further examined the versatility of the studied system. In addition to FloAc, we employed a different fluorescent comonomer Nile Blue acrylamide (NBAAm). The formulation was NAM:CDTPA:NBAAm:M2CMK, 420:1:1:1 in 4 m dioxane. For each formulation, a letter, a square of 100 μm, and a line of 5 μm were printed with a scanning speed of 100 mm s^−1^ and power 80 mW. Letters were printed with increasing layer spacing from 0.05 to 1.05 μm, while the layer spacing for square and lines was kept constant at 0.05 μm. The layer spacing refers to the vertical distance (*z*‐axis) between the *x*,*y*‐coordinate planes where the laser performs its scanning. The CLSM image of printed structures belonging to letters A to F is shown in **Figure** **5**a. While the squares belonging to letters A–F retain a well‐defined shape as in the previous experiment, the squares belonging to letters G–K exhibit visual indication of thermal polymer degradation (also see transmission image in Figure S8, Supporting Information) and were thus not examined further. This effect was caused by the increasing layer spacing of the letters, which led to an overall decreased printing time of the letters and corresponding squares, and subsequently shortened cooling time of the polymer, also probably in combination with shortened time for radical depletion by oxygen rediffusion, leading to higher local radical concentrations.

To determine the height of the brushes with the composition pNAM‐*co*‐NBAAm, AFM contact point (CP) images of the 2PRAFT polymerized brushes were recorded in air (Figure S4, Supporting Information) and sterile‐filtrated phosphate‐buffered saline (PBS) at pH 7.4 (Figure 5b). It should be emphasized that imaged polymer brushes represent nontypical AFM substrates where flexible polymer chains deform under the AFM tip. For this reason, the data analysis was optimized to obtain accurate thickness values. More details on AFM data evaluation are provided in Section S4 (Supporting Information). Generally, samples imaged in air showed lower brush thicknesses, 11.3 ± 1.6 nm compared to 15.9 ± 2.3 nm as measured in PBS solution. This effect is caused by the ability of hydrophilic pNAM brushes to swell in water and aqueous buffers. As seen in Figure 5b, brush‐containing regions appear homogeneously and are sharply delineated from the glass background. Additionally, round particles of various sizes are spread over all images taken, whereas they appear bigger and more frequently on images measured in air (Figure S5, Supporting Information). These are probably formulation residues since they could be successfully removed by additional soaking in PBS.

In order to further prove the livingness of the chain ends, end‐group labeling was performed using red fluorescent dye Alexa Fluor 647 (for further experimental details, see Figure S9 and S10 and Table S3, Supporting Information). The CLSM image (Figure S10, Supporting Information) confirms the successful end‐group labeling due to strong overall fluorescence in the red channel as well as the preservation of the polymer brush structures in the green channel. The insignificant difference in the red channel of only 1% (see Table S3, Supporting Information) confirms the presence of thiol groups on the grafted polymer chains. Based on reported results, the livingness of the chain ends was confirmed.

**Figure 6:**
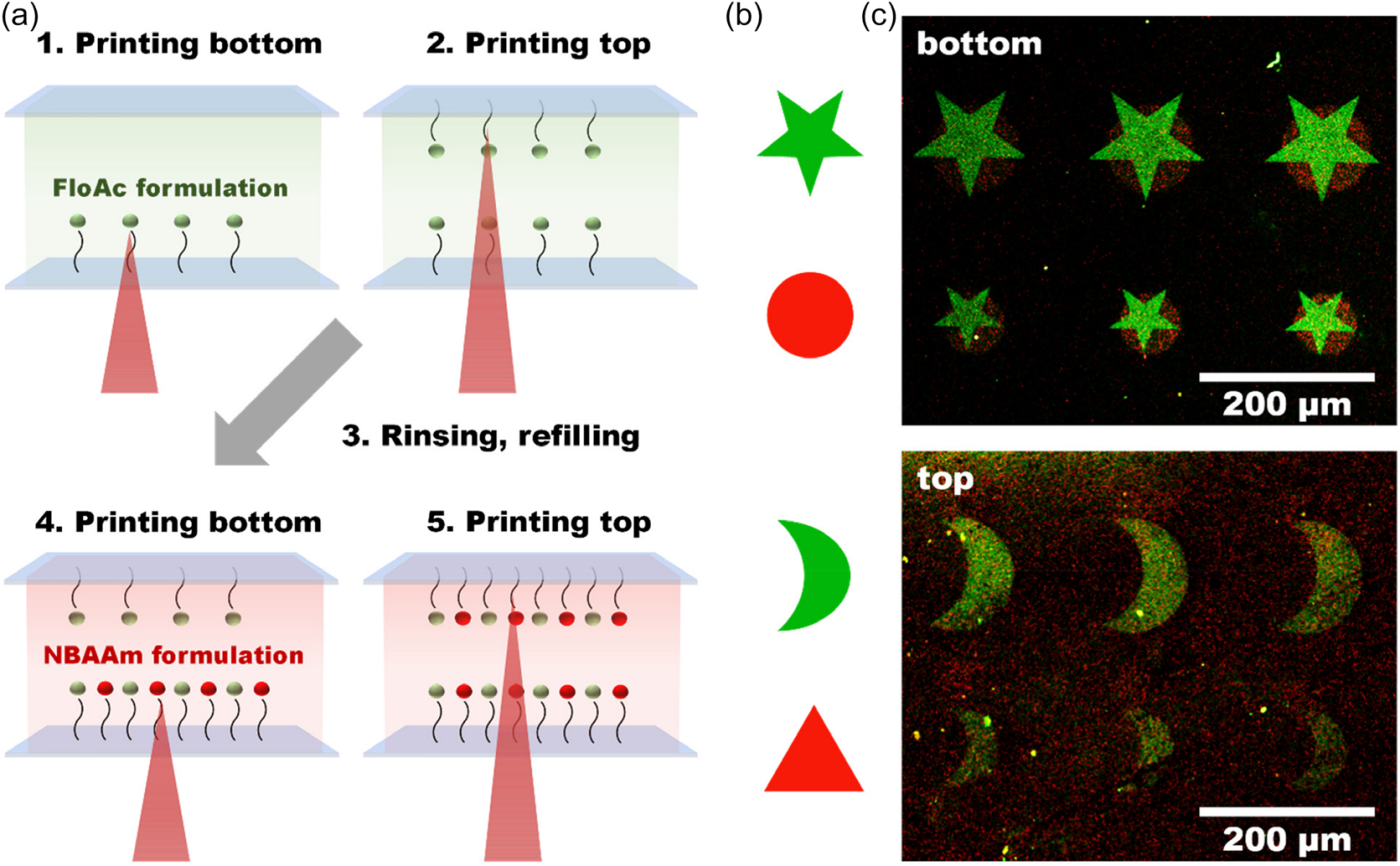
Patterned pNAM brushes printed onto parallel glass slides. a) Overview of the experimental design, b) Printed patterns, and c) CLSM images of cover glass (bottom) and microscope slide (top).

Patterned polymer brushes can find applications in various fields, one of them being microfluidic devices. To exhibit the potential of our system in this field, we designed a final printing experiment. The model setup was composed of two glass slides separated by a 1 mm glass spacer. The lower part consisted of a thin‐cover glass‐modified CDTPA. The upper part consisted of a 1 mm‐thick microscope slide, also modified with CDTPA. Such configuration represents a simplified model of a microfluidic channel. The gap between the two slides was filled with the formulation NAM:CDTPA:FloAc:M2CMK 420:1:1:0.25, 4 m in dioxane. The first pattern, a star array, was printed on the bottom cover glass, followed by the printing of the second pattern, a moon array, on the top microscope slide, through the existing previously printed pattern. Glass slides of the model microfluidic channel were rinsed with dioxane before the next printing step. The printing process was repeated with NBAAm formulation (NAM:CDTPA:NBAAm:M2CMK 420:1:1:0.25, 4 m in dioxane), with an array of circles printed on the bottom cover glass, and with an array of triangles printed on the top microscopic slide. The experimental design is depicted in **Figure** **6**a. After rinsing and disassembly, both slides were examined by CLSM. The resulting images are shown in Figure 6b. Both pattern types (green stars and red circles) are clearly visible on the bottom cover glass. With this experiment, we showed that 2PRAFT method can be used for subsequent modification of a surface by inks with different compositions, potentially containing different active substances. In addition, we showed that the washing step between the printing cycles does not disrupt the pattern formation, and the CDTPA modification remains stable. On the upper microscope slide, the moon array is visible, although less intense than on the bottom pattern. This is due to the necessary extension of the focal plane of the laser and the connected voxel elongation. However, this result proves the ability to print different patterns on adjacent surfaces, for example, the walls of a microfluidic channel. It should be emphasized that by using UV laser, such results are inherently unobtainable, since it would lead to the initiation of the polymerization along the light path, leading to patterning of both the lower and the upper surface simultaneously. On the upper slide, the red triangle pattern is not visible, although higher laser power was used for the printing to compensate for voxel elongation (120 mW in comparison to 80 mW). Possible interference of Nile Blue with the 2P initiator is not expected as the 2P cross section of the initiator is considerably higher (0.6 GM vs 269 GM at 800 nm).^[^ ^24^, ^29^ ^]^ To achieve better results, further optimization of the irradiation conditions would be necessary although a channel thickness of 1 mm—as demonstrated here—is most likely not necessary in the field (e.g., in a microfluidic chip).

## 3 Conclusion

To conclude, we described here 2PRAFT for the first time. Patterned brushes of pNAM with a height of around 10 nm were prepared by this method. Well‐defined printed structures with sharp edges down to 5 μm size were observed, which is much smaller than previously reported patterns via one‐photon direct laser writing.^[^ ^8^ ^]^ Moreover, here developed method enables creation of multicolored images and printing different patterns on adjacent surfaces (top and bottom glass slide stacks). Such surface modification method can find future applications in controlled cell adhesion, but also in modification of nonflat geometries—for example, microfluidic devices, among others.

## 4 Experimental Section

4.1

4.1.1

##### Materials

1‐Dodecanethiol, iodine, potassium *tert*‐butoxide, 4,4′‐azobis(4‐cyanovaleric acid) (ACVA), *N*‐Hydroxysuccinimide (NHS), Dulbecco's PBS, fluoresceine *o*‐acrylate (FloAc), and Nile Blue acrylamide (NBAAm) were purchased from Sigma–Aldrich and used as received. (3‐Aminopropyl)triethoxysilane (APTES) was purchased from ABCR and stored under an argon atmosphere at 8 °C. Naphthalene was purchased from Merck. 1‐Ethyl‐3‐(3‐dimethyl‐aminopropyl)carbodiimide EDCxHCl was purchased from ABCR, carbon disulfide was purchased from Loba Feinchemie, and Ivocerin was received from Ivoclar Vivadent. *N*‐Acryloylmorpholine (NAM, TCI chemicals) and triethylamine (TEA, Sigma–Aldrich) were distilled prior to the use. Chloroform‐*d* was purchased from Eurisotop. The solvents dioxane (JT‐Baker chemicals), acetone (Donau Chemie), dichloromethane DCM (Donau Chemie), petroleum ether PE (VWR), ethanol (Australco), tetrahydrofuran THF (VWR), and ethyl acetate (Fluka) were distilled prior use.

##### Precursor Syntheses: Synthesis of Bis‐(Dodecylsulfanylthiocarbonyl) Disulfide

The synthesis of bis‐(dodecylsulfanylthiocarbonyl) disulfide was performed according to the procedure described by Moad et al.^[^ ^30^ ^]^ with slight modifications. 1.06 equiv. of potassium *tert*‐butoxide (4.24 g, 38 mmol) was dissolved in a PE/THF (120 mL/30 mL) mixture and cooled down to 5 °C with an ice/water bath. 1 equiv. of 1‐dodecanethiol (7.27 g; 36 mmol) was added dropwise and the solution became a white emulsion. This emulsion was further stirred for 30 min at 5 °C and subsequently 1.06 equiv. of carbon disulfide (2.85 g, 37 mmol) was added dropwise to the mixture. The formed yellow foam was stirred for 4 h at room temperature, then 0.53 equiv. of iodine (9.54 g, 38 mmol) dissolved in THF (20 mL) was added dropwise, and the solution was stirred for a further 12 h.

The dark brown mixture was completely dissolved in PE (500 mL) and the organic phase was extracted with aqueous thiosulfate solution until the color changed to bright orange, followed by washing with saturated sodium chloride solution. Afterward, the organic phase was dried over sodium sulfate and filtered, and the solvent was removed in vacuo. The product was obtained as orange oil, which crystallizes at lower temperatures to a wax‐like orange solid and was isolated in a yield of 85% of theory (8.46 g).

^1^H‐NMR (400 MHz, CDCl_3_): *δ* [ppm] 0.88 (t; 6 H; *J* = 7.05 Hz; (‐S‐…‐C(12)H_3_)_2_), 1.19–1.47 (m; 36 H; (‐S‐…C(3)‐(11)H_2_‐)_2_), 1.69 (quint; 4 H; *J* = 7.80 Hz (‐S‐…C(2)H_2_‐)_2_), 2.68–0.3 (m; 4 H; *J* = 7.42 Hz; (‐S‐C(1)H_2_‐)_2_)

##### Precursor Syntheses: Synthesis of Cyano‐4‐(((Dodecylthio)‐Carbonothioyl)‐Thio)‐Pentanoic Acid (CDTPA)

The synthesis of cyano‐4‐(((dodecylthio)‐carbonothioyl)‐thio)‐pentanoic acid was performed according to Moad et al.^[^ ^30^ ^]^ and Rizzardo et al.^[^ ^31^ ^]^ 1 equiv. of bis‐(dodecylsulfanylthiocarbonyl) disulfide (5 g, 9 mmol) was dissolved in ethyl acetate (50 mL) and the solution was degassed with argon for 15 min. The mixture was refluxed under an inert atmosphere and 1.7 equiv. of 4,4′‐azobis (4‐cyanovaleric acid) (4.3 g, 15.3 mmol) was slowly added under argon counter flow. The mixture was refluxed for a further 12 h and monitored by TLC (Rf = 0.24; PE:EE = 1:1).

The solvent was removed via vacuum distillation and the residue was purified via liquid column chromatography over silica (1.4 g crude product; 120 g silica; PE:EE = 1:1) to yield the product as a yellow solid in a yield of 31 % of theory (2.29 g).

^1^H‐NMR (400 MHz, CDCl3): *δ* [ppm] 0.88 (t; 3 H; *J* = 7.06 Hz; (‐S‐…‐C(12)H_3_)), 1.19–1.45 (m; 18 H; (‐S‐…C(3)‐(11)H_2_‐)), 1.69 (quint; 2 H; *J* = 7.43 Hz (‐S‐…C(2)H_2_‐)), 1.88 (s; 3 H; (‐S‐C*‐CH_3_)), 2.38–2.74 (m; 4 H; (‐C*‐C(2)’H_2_‐C(3)’H_2_‐)), 3.3 (t; 2 H; *J* = 7.48 Hz; (‐S‐C(1)H_2_‐))

##### Precursor Syntheses: Synthesis of 1‐Cyano‐4‐(2,5‐Dioxo‐1‐Pyrrolidinyloxy)‐1‐Methyl‐4‐ Oxobutylthiotridecanedithioate

1 equiv. of CDTPA (500 mg, 1.24 mmol) was dissolved in dry DCM (10 mL) and stirred at 0 °C in an ice/water bath. Subsequently 1.2 equiv. of EDCxHCl (285 mg, 1.49 mmol) dissolved in dry DCM (10 mL) was added dropwise to the solution. The solution was stirred at 0 °C in an ice/water bath for 1 h. To the cooled reaction mixture, 1.2 equiv. of NHS (171 mg, 1.49 mmol) dissolved in absolute DCM (10 mL) was added dropwise, the solution was stirred for 18 h at room temperature, and the conversion was monitored by TLC (Rf = 0.68; petroleum:ethyl acetate = 1:1).

The solvent was removed in vacuo and the residue was redissolved in ethyl acetate (50 mL). The organic phase was extracted with saturated NaHCO_3_ solution (2 × 25 mL), deionized water (1 × 25 mL), and brine (1 × 25 mL). The resulting organic phase was dried over sodium sulfate and the solvent was removed in vacuo. The product was isolated by recrystallization from dry acetone as a yellow solid in a yield of 88% of theory (511 mg).

^1^ H‐NMR (200 MHz, CDCl_3_, *δ* [ppm]): 0.88 (*t*; 3 H; *J* = 6.87 Hz; (‐S‐…‐C(12)H_3_)), 1.18–1.48 (m; 18 H; (‐S‐…‐C(3)‐(11)H_2_‐)), 1.59–1.79 (m; 2 H; (‐S‐…‐C(2)H_2_‐)), 1.89 (s; 3 H; (‐S‐C*‐CH_3_)), 2.47–2.94 (m; 4 H; (‐C*‐C(2)’H_2_‐C(3)’H_2_‐)), 2.85 (s; 4 H; (‐CH_2_‐CH_2_‐ NHS ring), 3.3 (t; 2 H; *J* = 7.47 Hz; (‐S‐C(1)H_2_‐))

##### Surface Modifications: Sample Preparation and Vapor Deposition of Aminopropyl Triethoxysilane (APTES) on Silicon Wafer/Glass Substrates

Square silicon wafer samples with dimensions 2 × 2 cm were cut by a diamond tip from a silicon wafer with a diameter 10 cm, thickness 525 ± 25 μm, Type P/Bor, Orientation <100> (Si‐Mat—Silicon Materials e.K., Kaufering, Germany). Glass coverslips were used in the purchased size 24 × 60 mm, thickness ≈0.17 mm (#1.5) (Menzel Gläser, Braunschweig, Germany). Prior to modification, the samples were thoroughly rinsed with water, acetone, and toluene (3×) and dried under an argon stream. After the solvent cleaning step, the samples were further treated with a UV oven (UV Clean, model 135500, Boekel Scientific, Pennsylvania, USA) for 10 min to activate the surface. The cleaned and activated samples were put inside a tube‐shaped glass reactor with an extra reservoir equipped with a vacuum valve for vapor‐phase APTES deposition. For more experimental details, please see SI. After APTES deposition, the samples were removed, characterized via ellipsometry (Si wafer), and immediately used for the next modification step.

##### Surface Modifications: Modification of Amino‐Functionalized Surfaces with 1‐Cyano‐4‐(2,5‐Dioxo‐1‐Pyrrolidinyloxy)‐1‐Methyl‐4‐Oxobutylthiotridecanedithioate

For the preparation of modified glass cover slides, 1‐cyano‐4‐(2,5‐dioxo‐1‐pyrrolidinyloxy)‐1‐methyl‐4‐oxobutylthiotridecanedithioate (NHS‐CDTPA) (40 mg; 0.1 mmol) was dissolved in dry DCM (40 mL) and TEA (12 μL; 0.1 mmol) was added immediately. The solution was filtered via a syringe filter into a Schlenk‐tube and the prior amino‐functionalized glass slides (4 samples per 40 mL of reaction mixture) were immersed into the solution overnight at room temperature. Then, the substrates were cleaned by thorough rinsing with absolute DCM and dried under an argon stream. As a control, 1 cm^2^ Si wafer sample was added to the cover glass samples to allow measurement of the thickness by ellipsometry.

The silicon wafers were modified analogously; for more details, see the Supporting Information.

##### Surface Modifications: General Procedure for Visible Light‐Induced Grafting from RAFT Polymerization from CDTPA Functionalized Substrates

The composition of photosensitive formulation differs according to the desired outcome (i.e., polymer chain length, monomer:CTA:initiator ratio). As an example, the formulation 60 kDa pNAM with NAM:CDTPA:PI ratio 420:1:0.25 contained 500.2 mg of NAM (3.54 mmol, 420 equiv.), 3.4 mg of CDTPA (0.008 mmol, 1 equiv.), and 0.6 mg of Ivocerin (0.002 mmol, 0.24 equiv.) in dioxane (0.9 mL).

The NHS‐CDTPA modified Si‐wafers were coated with a 10 μL drop of the prior degassed (15 min argon) photosensitive formulation under argon atmosphere and covered with a 150 μm thick glass coverslip. The substrate was put inside a vessel on a microscope slide under constant argon stream. A light source (Exfo OmniCure S200 broadband Hg lamp with a 400–500 nm filter) was fixed at a defined distance (2.5 cm) from the substrate to have a constant intensity of 28.8 mW cm^−2^ at the substrate surface (measured with an Ocean Optics USB 2000+ spectrometer). The substrates were irradiated for selected time intervals (120, 360, 720 s) and subsequently immersed in THF (1.5 mL) to collect free polymer samples for GPC measurement. After further cleaning with dioxane, the wafers were analyzed via ellipsometry.

##### Surface Modifications: Patterning via Two‐Photon‐Induced RAFT Grafting from Polymerization on Glass Substrates

In order to adapt the photosensitive formulations for 2PP, the one‐photon initiator Ivocerin was replaced by (2E,6E)‐2,6‐bis(4‐(dimethylamino)benzylidene)‐4‐methylcyclohexanone (M2CMK), an efficient 2P initiator.^[^ ^24^ ^]^ Formulation 1 contained CDTPA (3.4 mg; 0.008 mmol), NAM (500 mg; 3.54 mmol), fluorescein‐*o*‐acrylate (FloAc) (3.3 mg; 0.008 mmol), and M2CMK (3.1 mg; 0.008 mmol) in dioxane (0.9 mL). Formulation 2 contained CDTPA (3.4 mg; 0.008 mmol), NAM (503 mg; 3.56 mmol), Nile Blue acrylamide (NBAAm) (3.0 mg; 0.007 mmol), and M2CMK (3.0 mg; 0.008 mmol) in dioxane (0.9 mL). FloAc and NBAAm were added as fluorescence labeling.

Glass coverslips were aminofunctionalized via APTES vapor deposition and further modified with NHS‐CDTPA (as described before). The substrates were scratched to label the printing area. To perform the experiment, the modified glass coverslips were fixed on the stage of the 2PP printer, and two 1 mm thick glass spacers were placed beside the printing area. A 10 μL drop 2P‐sensitive formulation was placed on the marked area between the spacers and was covered by another glass coverslip, to prevent evaporation. The used 2PP printer is a custom setup based on a tunable femtosecond near‐infrared laser (MaiTai eHP DeepSee, Spectra‐Physics), with 70 fs pulse duration after the microscope objective (uPlanXApo, 10x, 0.4 NA, Olympus), and 80 MHz repetition rate.^[^ ^15^ ^]^ The laser wavelength used for printing experiments was 800 nm. After calibration of the device to the focal plane, the printing process was started according to predefined printing patterns (letters A–K, squares, lines). The whole patterns were printed with different laser writing speeds of 1000 and 100 mm s^−1^, with laser intensity variations from 50 to 150 mW, lateral line distance of 1.5 μm, and z‐gap distance from 0.05 to 1.1 μm (layer separation). After the printing process, the substrates were cleaned with dioxane and analyzed via a laser scanning microscope (Zeiss LSM 800 Airyscan).

##### Surface Modifications: Patterning via Two‐Photon‐Induced RAFT Grafting from Polymerization on Glass Substrate Stacks

Glass coverslip (thickness ≈0.17 mm) and microscope slide (thickness 1 mm) were amino‐functionalized via APTES vapor deposition and further modified with NHS‐CDTPA (as described before). The substrates were scratched to label the printing area. Modified glass coverslips were fixed on the stage of the 2PP printer, and two 1 mm‐thick glass spacers were placed beside the printing area. Onto the glass spacers, the modified microscope slide was placed. A 20 μL drop of 2P‐sensitive formulation 1 (containing fluorescein‐*o*‐acrylate) was injected using a syringe on the marked area between the spacers. The printing was performed with the previously described setup. On the bottom surface (glass coverslip), an array of stars of different sizes was printed using 80 mW laser intensity and a scanning speed of 100 mm s^−1^. On the top surface (microscope slide), an array of half‐moons of different sizes was printed, using scanning speed 100 mm s^−1^ and 120 mW laser intensity, to compensate for voxel elongation. The glass surfaces were washed with dioxane and dried with argon stream and the printing cycle was repeated with formulation 2 (Nile Blue acrylamide). On the bottom surface (glass coverslip), an array of circles of different sizes was printed using 80 mW laser intensity and a scanning speed of 100 mm s^−1^. On the top surface (microscope slide), an array of triangles of different sizes was printed using scanning speed 100 mm s^−1^ and 120 mW laser intensity.

##### Analytical Methods: NMR Spectroscopy

NMR spectra were recorded on Bruker Avance spectrometers at 400/600 MHz for ^1^H‐ and at 100/200 MHz for ^13^C spectra and on a Bruker DPX‐200 FT spectrometer at 200 MHz for ^1^H‐ and 50 MHz for ^13^C spectra. Ten milligrams of the sample was dissolved in CDCl_3_. Spectra of photosensitive compounds were measured in brown‐glass NMR tubes. Data for ^1^H‐NMR are reported as follows: chemical shift (*δ*) in units of parts per million (ppm) from tetramethylsilane using the residual nondeuterated solvent signal *δ* = 7.26 ppm as an internal reference. Analysis of the spectra was performed with the software TopSpin (2.1) by Bruker.

##### Analytical Methods: Gel Permeation Chromatography

GPC measurements were performed on a Malvern Viscotek TDA system equipped with a Viscotek SEC MALS 9 light scattering detector, a Viscotek TDA 305‐021 refractive index and viscosity detector, and a UV Detector Module 2550 for TDA 305. The samples were prepared as syringe‐filtered 2−4 mg mL^−1^ THF solutions spiked with 0.5 mg mL^−1^ butylhydroxytoluene (BHT) as a flow rate marker. Separation was conducted through three consecutive PSS SDV columns (100, 1000, 100 000 Å) with PSS SDV precolumn, using THF as eluent at a flow rate of 0.8 mL min^−1^. Conventional standard calibration was performed with PSS between 470 and 44 000 g mol^−1^. The recorded spectra were analyzed with the software OmniSEC Version 05.12.461 by Malvern.

##### Analytical Methods: Atomic Force Microscopy

The thickness of dry and hydrated polymer brushes prepared via 2PRAFT was examined by AFM. After 2PRAFT, the modified samples were thoroughly washed with dioxane and stored under vacuum until AFM measurements were performed. To reduce the risk of sample damage, brushes‐carrying glass slides were glued into glass Petri dishes using epoxy glue (2 K Epoxidkleber, Uhu Plus). Prior to AFM imaging, the Petri dishes were rinsed at least twice with ethanol and dried using nitrogen and additionally rinsed at least twice with PBS for imaging in PBS.

AFM experiments were performed on a NanoWizard ULTRA SpeedA AFM system (JPK Instruments AG, Berlin) equipped with an inverted optical microscope (Axio Observer.D1, ZEISS). Polymer Brushes were imaged using a 4XC‐NN rectangular cantilever (3.45 N m^−1^ measured spring constant; μMasch, OPUS) equipped with a sharp tip (nominal tip radius < 7 nm). The cantilever spring constant was calibrated with the thermal noise method^[^ ^32^ ^]^ and the deflection sensitivity was obtained by performing 32 force‐distance measurements on the glass surface next to the sample, as previously described.^[^ ^33^ ^]^ To investigate pNAM brushes, a 30 × 30 μm region of interest was scanned in quantitative imaging mode (QI, JPK) with a spatial resolution of 256 × 256 and a maximum applied force within 10–20 nN range. More details on the AFM method are available in Section S2 (Supporting Information—Atomic force microscopy, Figure S4, Supporting Information).

##### Analytical Methods: X‐Ray Photoelectron Spectroscopy

The surface chemical composition of the control sample, each modification step (Si wafer, APTES, CDTPA), and the final polymer brush pNAM*‐co*‐FloAc 60 kDa were determined by XPS. For XPS analysis, Thermo Scientific K‐Alpha XPS system (Thermo Fisher Scientific, UK) equipped with a microfocus, monochromatic Al Kα X‐ray source (1486.68 eV) was used. The constant analyzer energy mode with the pass energy of 200 eV was applied for obtaining the survey scan, and narrow regions were collected with the pass energy of 50 eV. Flood gun was used for the charge compensation. Thermo Scientific Avantage software (version 5.9925, Thermo Fisher Scientific, UK) was used for digital acquisition and data processing. A Powell fitting algorithm with 100 iterations was used to fit the high‐resolution XPS peaks. The line shapes were the product of Lorentz/Gauss mix and were fixed at *L*/*G* = 30% and a smart background was used. The repeatability of the results was examined by evaluation of three separate wafers subjected to surface modification independently. The final chemical composition is expressed as mean ± SD of these measurements and is listed in Table S1, Supporting Information.

##### Analytical Methods: Ellipsometry

Brush thickness measurements were carried out using a Sentech SE 500adv equipped with a He‐Ne light source and a rotating analyzer. The used wavelength was 632.8 nm. Data analysis was performed with the supplied instrument software SE400advanced 2.16, which uses the McCracking algorithm. The used optical constants were Si (*n* = 3.865, *k* = 0.02), SiO_2_ (*n* = 1.465, *k* = 0), and organic layer (*n* = 1.5, *k* = 0). The measurements were performed at six different spots on each sample's surface. The data are presented as mean ± standard deviation, while the outliers were excluded using the Grubbs outlier test (Besh Stat, free open‐source statistical add‐in for MS Excel, http://beshstat.eu/).

## Conflict of Interest

The authors declare no conflict of interest.

## Author Contributions

**Stefan Helfert**: Investigation (equal); Methodology (equal); Validation (equal); Writing—original draft (equal). **Tommaso Zandrini**: Data curation (equal); Formal analysis (equal); Investigation (equal); Methodology (equal); Validation (equal); Visualization (equal); Writing—review and editing (supporting). **Andreas Rohatschek**: Data curation (equal); Investigation (equal); Validation (equal); Visualization (equal); Writing—original draft (supporting). **Manuel Rufin**: Investigation (supporting); Visualization (supporting); Writing—original draft (supporting). **Peter Machata**: Data curation (supporting); Formal analysis (supporting); Investigation (supporting); Methodology (supporting); Validation (supporting); Visualization (supporting); Writing—original draft (supporting). **Anna Zahoranová**: Conceptualization (lead); Data curation (equal); Formal analysis (equal); Funding acquisition (equal); Investigation (lead); Methodology (lead); Validation (equal); Visualization (equal); Writing—original draft (lead); Writing—review and editing (equal). **Orestis G. Andriotis**: Formal analysis (supporting); Investigation (supporting); Methodology (supporting); Validation (supporting); Visualization (supporting); Writing—original draft (supporting). **Philipp J. Thurner**: Methodology (equal); Supervision (equal); Validation (supporting); Writing—review and editing (supporting). **Aleksandr Ovsianikov**: Funding acquisition (supporting); Methodology (supporting); Resources (supporting); Supervision (supporting); Validation (supporting); Writing—review and editing (supporting). **Robert Liska**: Resources (supporting); Supervision (supporting); Writing—review and editing (supporting). **Stefan Baudis**: Conceptualization (lead); Formal analysis (supporting); Funding acquisition (lead); Investigation (supporting); Methodology (equal); Project administration (lead); Resources (lead); Supervision (lead); Validation (supporting); Visualization (equal); Writing—original draft (equal); Writing—review and editing (equal).

## Supporting information

Supplementary Material

## Data Availability

The data that support the findings of this study are available from the corresponding author upon reasonable request.
