# Supplementary material for: Micropatterning of Confined Surfaces with Polymer Brushes by Two‐Photon‐Initiated Reversible Addition–Fragmentation Chain‐Transfer Polymerization
Source: Small Sci. 2024 Nov 21;5(1):2400263. doi: 10.1002/smsc.202400263 (PMC11934993; doi:10.1002/smsc.202400263)
Supplement: Supplementary file 1 — Supplementary Material [file SMSC-5-2400263-s001.pdf]

# Supporting information

# Micropatterning of confined surfaces with polymer brushes by two-photon-initiated RAFT polymerization

*Stefan Helfert,[1,6] Tommaso Zandrini,[3,6] Andreas Rohatschek,[2,4,6] Manuel Rufin,[4,6] Peter Machata,[5] Anna Zahoranová,[1,2,6]* Orestis G. Andriotis,[4,6] Philipp J. Thurner,[4,6] Aleksandr Ovsianikov,[3,6] Robert Liska,[1,6] Stefan Baudis[1,2,6]**

1. Institute of Applied Synthetic Chemistry, TU Wien, Getreidemarkt 9/163MC, Vienna 1060, Austria
2. Christian Doppler Laboratory for Advanced Polymers for Biomaterials and 3D Printing
3. Institute of Materials Science and Technology, TU Wien, Getreidemarkt 9/308, Vienna 1060, Austria
4. Institute of Lightweight Design and Structural Biomechanics, TU Wien, 1060 Vienna, Austria
5. Department of Composite Materials, Polymer Institute of the Slovak Academy of Sciences, Dúbravská cesta 9, 84541, Bratislava, Slovakia
6. Austrian Cluster for Tissue Regeneration, Vienna, Austria

*Corresponding authors:

A. Zahoranová (anna.zahoranova@tuwien.ac.at)

S. Baudis (stefan.baudis@tuwien.ac.at)

**Content**

1. Materials and methods ..... 2
2. Brush thickness and molecular weight ..... 2
3. Kinetics investigation of visible light induced RAFT polymerization ..... 3
4. Atomic force microscopy (AFM) ..... 5
5. X-ray photoelectron spectroscopy (XPS) ..... 7
6. Two-coloured image of end-group cleaved and labeled pNAM-pFloAc brushes on glass surface ..... 10
7. References ..... 11

# 1. Materials and methods

*Vapor deposition of aminopropyl triethoxysilane (APTES) on silicon wafer/glass substrates*

The cleaned and activated samples (wafers/glass slides) were put inside a tube-shaped glass reactor with an extra reservoir equipped with a vacuum valve for vapor-phase APTES deposition. The apparatus was placed into a pre-heated Kugelrohr glass oven (Büchi Labortechnik AG, Flawil, Switzerland) at 115 °C. The extra reservoir outside of the oven was filled with APTES (0.1 mL) and the whole apparatus was evacuated to 5 mbar. When the target pressure was reached, the valve was closed and APTES in the reservoir was heated to its boiling point with a heat gun for 5 s. After this heat pulse, the samples were baked in the heated reactor for further 30 min. The apparatus was then purged with argon and allowed to cool down. After the cooling, the samples were removed, characterized via ellipsometry (Si wafer) and immediately used for the next modification step.

*Modification of amino-functionalized Si wafers with 1-cyano-4-(2,5-dioxo-1-pyrrolidinyloxy)-1-methyl-4-oxobutylthiotridecanedithioate (NHS-CDTPA)*

The cut wafers were placed separately into glass vials filled with 2 ml of filtered reaction of the mixture (1 mg mL-1 of NHS-CDTPA, 0.3 μL mL 1 TEA in DCM) per Si wafer, stored overnight at 8 °C. After cleaning, the thickness of the coating was measured via ellipsometry.

# 2. Brush thickness and molecular weight

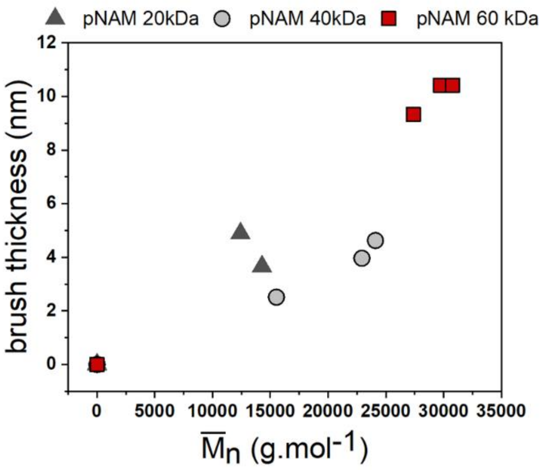


**Figure S1.** Brush thickness (as measured by ellipsometry) vs $\overline{M_n}$ (as measured by GPC) of polymers with different targeted $\overline{M_n}$ values.

A rough estimation of the brush formation achieved, can be obtained according to Mutlutürk *et al.*[1] The grafting density σ could be obtained from the UV/Vis approaches by calculating the slope in brush thickness formation (Figure 2b) vs. molecular weight from monomer conversion derived from the kinetical experiment (Figure S2) employing the following equation.

$$\sigma = \frac{h * \varrho * N_A}{M_n * 10^{21}}$$

σ: grafting density, chains·nm$^{-2}$;

h: brush thickness, nm;

ρ: polymer density, 1.074 g·cm$^{-3}$, ref.[1]

$N_A$: Avogadro's number, $6.02 \cdot 10^{23}$ mol$^{-1}$

$M_n$: number-average molecular weight derived from $^1$H-NMR, g·mol$^{-1}$

$10^{21}$: factor of nm$^3$·cm$^{-3}$

Measurement of the radius of gyration for pNAM polymers in water derived from RAFT polymerization and static light scattering was previously described[2] and by using the calculated value for $\sigma$ = 0.14 chains·nm$^{-2}$, a reduced tethered density $\Sigma = \sigma \pi R_g^2$ of $\Sigma >> 1$ could be determined, showing according to Brittain and Minko[3] that brushes in the highly stretched regime could be obtained via this grafting-from method. It should be noted that based on static and dynamic light scattering experiments, radius of gyration $R_g$ in used experimental conditions (measured in miliQ water in polymer concentration 1.5 mg/mL) was 14.63 nm, which suggests presence of worm-like aggregates. Unfortunately, also by changing the polymer concentration and the solvent to PBS, we were unable to obtain lower values, which would suggest the presence of single polymer chains.

## 3. Kinetics investigation of visible light induced RAFT polymerization

A formulation of CDTPA (5 mg; 0,013 mmol), NAM (500 mg; 3.54 mmol), Ivocerin (0.61 mg; 0.002 mmol) and naphthalene (100 mg; 0.78 mmol) as internal standard in dioxane (3.6 mL) was prepared. The solution was degassed with argon for 15 min and the polymerization was performed in a 25 mL flask photoreactor under continuous stirring. A 400 – 500 nm light source was fixed to a constant distance (2.5 cm) to the interface of the formulation and the intensity for the distance was determined via ocean optics (28.8 mW.cm$^{-2}$). The reaction was monitored via $^1$H-NMR and GPC during an irradiation period of 900 s (Figure S1 and S2).

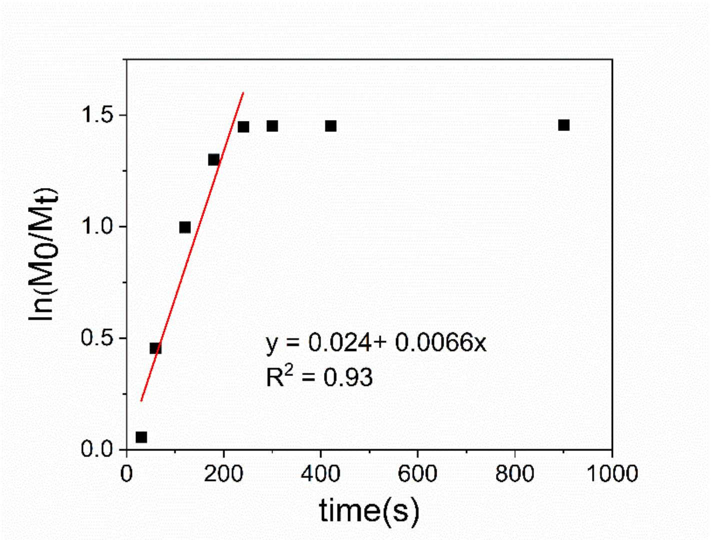


**Figure S2.** PhotoRAFT kinetics of NAM in dioxane

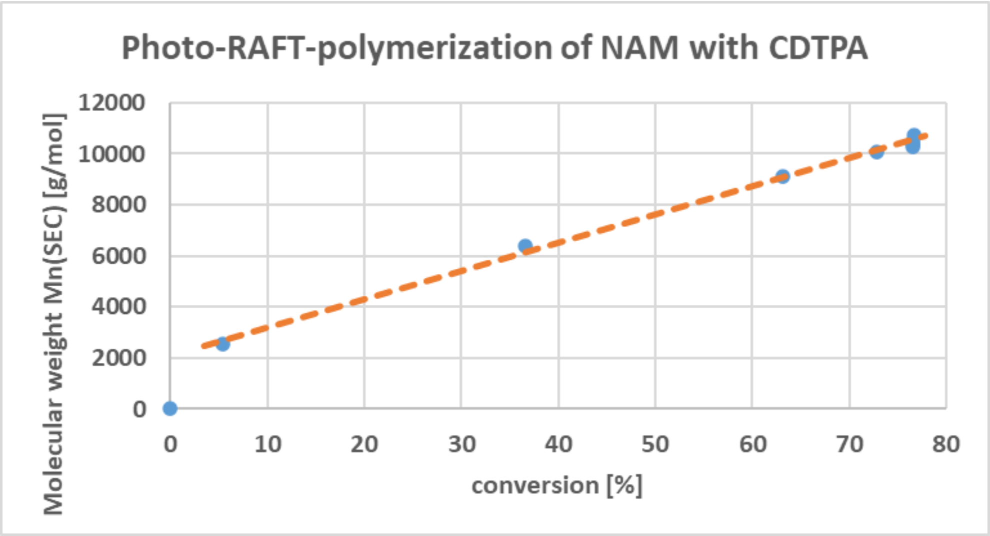


**Figure S3.** Dependence of $\overline{M_n}$ (as measured by GPC) on conversion (%) of NAM polymerization in dioxane

## 4. Atomic force microscopy (AFM)

In Quantitative Imaging mode (QI™, JPK), the AFM performs a series of fast indentations in a predefined, evenly spaced grid of the target resolution. For the indentations the AFM head moves down on one of the grid points with a constant speed until a particular setpoint force is detected. By default, the height of the AFM head at the setpoint force is used as a pixel value for imaging. For surfaces with a contact stiffness far higher than the cantilever spring constant, this accurately depicts the morphology of the underlying sample, barring convolution effects. However, the morphology becomes distorted if substantial deformation occurs in the sample during indentation. This is especially the case for the polymer brushes probed in this work. Since they are loosely connected to the solid support, even the small setpoint forces exerted by the cantilever will deform and press them down substantially.

To accurately depict the brushes height, we deployed contact point imaging, a technique where for every indentation curve of the QI image the point of first contact of the cantilever with the sample is determined.[4]

For data analysis, a custom-built Matlab® script (2021a, The MathWorks Inc., Natick, Massachusetts, USA) was used. Briefly, two different algorithms were applied to account for the differences caused by the media used for imaging (air, PBS). In PBS, the force-distance curves are perfectly flat up until the moment of first cantilever tip to surface contact, at which point the force monotonically increases. Here, a threshold principle was used where the contact point is determined as the first data point exceeding six times the standard deviation of the mean numerical derivative of the smoothed force data points.

In air, on the other hand, substantial attractive electrostatic forces act from the surface towards the cantilever, leading to the so-called ‘snap-in’ effect, a small negative deflection peak just before the actual tip to surface contact. Here, the contact point is considered as the point crossing the zero-force baseline after the snap-in peak and the corresponding cantilever head height is interpolated from the adjacent points around the zero-crossing.

In the next step, regions of interest on the brushes and the surrounding background (glass) are chosen and the corresponding data is read out. An overlay of the chosen regions with the corresponding AFM image can be seen in Figure S3A)

Since distribution of data derived from polymer brush and glass is reasonably approximated by normal distributions, quantile-quantile plots are used to separate sample data from printing artefacts. See Figure S3B) for the quantile-quantile plot of a brush segment. Finally, statistical properties (median, mean, standard deviation) of the selected data points are determined and the brush related data is corrected by the received values for the glass background.

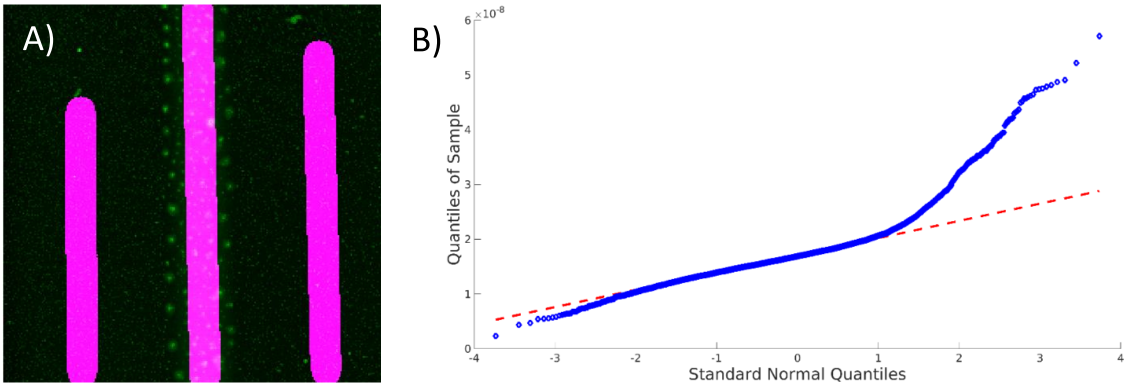


**Figure S4.** An example of segment-AFM-image overlay and quantile-quantile plot as received during brush height analysis. A) The pink lines show the regions of interest chosen on the AFM CP images and used for further analysis. B) Quantile-Quantile plot of a brush region of interest. Sample Quantiles are plotted against standard normal quantiles with sample data points shown as blue diamonds and normal distribution shown as red dots. The overlap region indicates normal distributed data points.

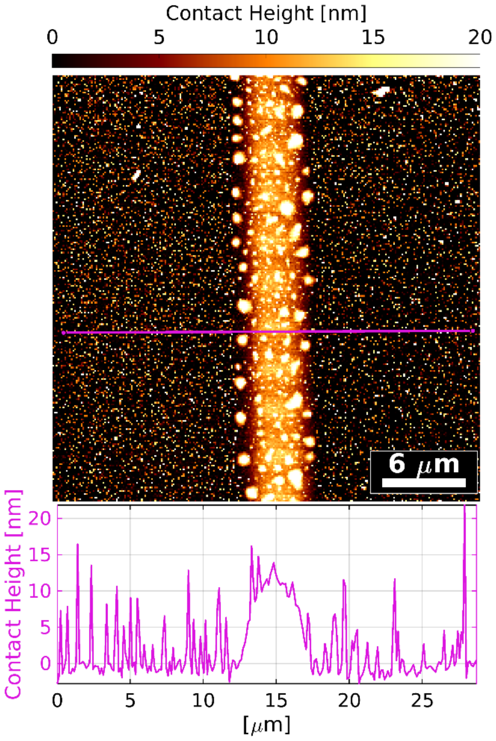


**Figure S5.** 30 µm x30 µm AFM CP image of 2PRAFT polymerized 5 µm wide line of. pNAM-*co*-pNBAAm polymer brushes imaged in air prior rinsing and measuring in PBS. Corresponding cross section of a selected region (purple line) on each image, are shown.

## 5. X-ray photoelectron spectroscopy (XPS)

**Table S1**. Apparent surface composition of APTES, CDTPA and pNAM60kDa as determined from high resolution XPS spectra.

| Sample (stoichiometry by XPS) | Surface chemical composition (at.%) | | | |
|---|---|---|---|---|
| | **C1*s*** | **O1*s*** | **N1*s*** | **Si2*p*** |
| | $sp^3$ | C=O | C-N | Si |
| | C-O (CN) | C-O (Si-O) | $C\text{-}N^+$ | $SiO_2$ |
| | C=O | O*-(C=O)-C | | |
| | O-C=O | | | |
| | N-C=O | | | |
| **Si wafer** | **3.7** | **38.0** | **-** | **58.4** |
| (reference) | 1.4 | 2.2 | - | 46.5 |
| | 0.8 | 33.9 | - | 11.8 |
| | 0.8 | 1.8 | | |
| | 0.7 | | | |
| | 0 | | | |
| **APTES** | **14.9 ± 0.4** | **33.8 ± 0.2** | **3.4 ± 0.2** | **48.0 ± 0.5** |
| | 7.6 ± 0.4 | 2.3 ± 0.3 | 2.0 ± 0.1 | 35.5 ± 0.6 |
| | 5.1 ± 0.2 | 30.4 ± 0.2 | 1.4 ± 0.1 | 12.4 ± 0.2 |
| | 1.5 ± 0.1 | 1.0 ± 0.1 | | |
| | 0.7 ± 0.2 | | | |
| | 0 | | | |
| **CDTPA** | **18.6 ± 2.0** | **32.3 ± 0.6** | **5.3 ± 0.8** | **43.9 ± 2.2** |
| | 9.8 ± 1.5 | 5.2 ± 1.1 | 4.5 ± 0.9 | 32.4 ± 2.2 |
| | 5.1 ± 0.3 | 26.2 ± 1.6 | 0.8 ± 0.1 | 11.5 ± 0.1 |
| | 1.4 ± 0.3 | 0.9 ± 0.1 | | |
| | 1.3 ± 0.6 | | | |
| | 0.9 ± 0.2 | | | |
| **pNAM60kDa** | **65.0 ± 2.3** | **20.7 ± 0.4** | **9.9 ± 0.2** | **4.5 ± 1.8** |
| | 21.6 ± 2.7 | 9.6 ± 0.5 | 9.3 ± 0.2 | 2.4 ± 1.3 |
| | 30.7 ± 4.7 | 10.7 ± 0.2 | 0.6 ± 0.2 | 2.1 ± 0.5 |
| | 6.4 ± 2.3 | 0.4 ± 0.2 | | |
| | 0.8 ± 0.2 | | | |
| | 5.4 ± 1.5 | | | |

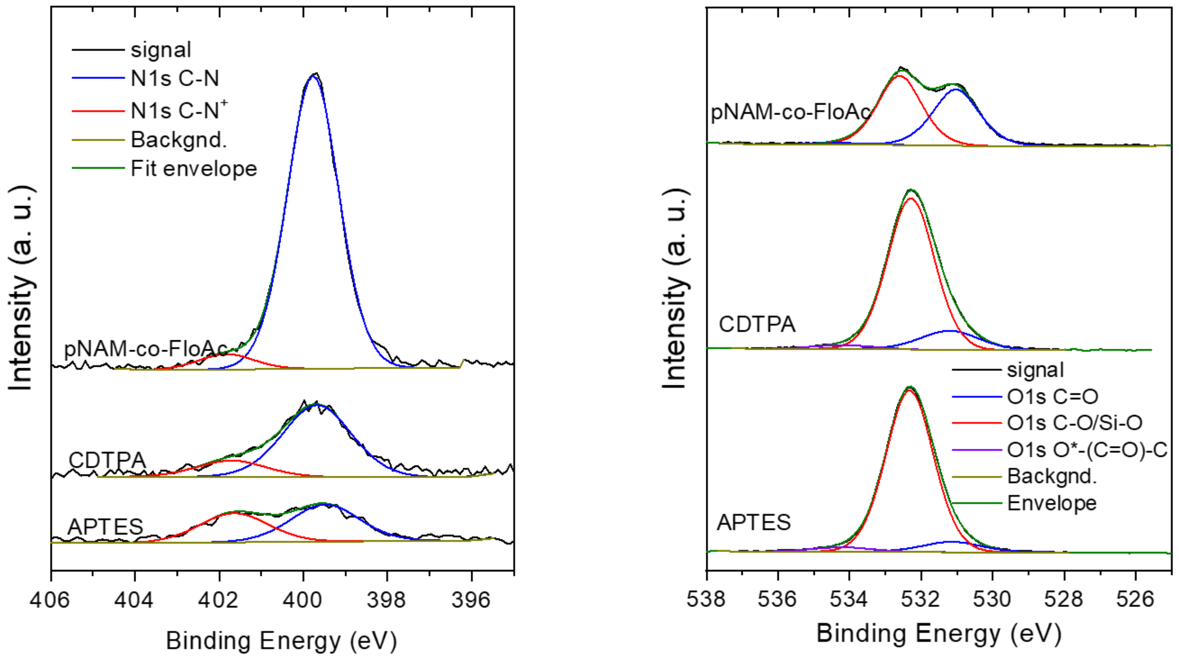


**Figure S6.** XPS spectroscopy of APTES, CDTPA and pNAM-*co*-FloAc 60kDa - (left) N1s spectra after deconvolution and (right) O1s spectra after deconvolution.

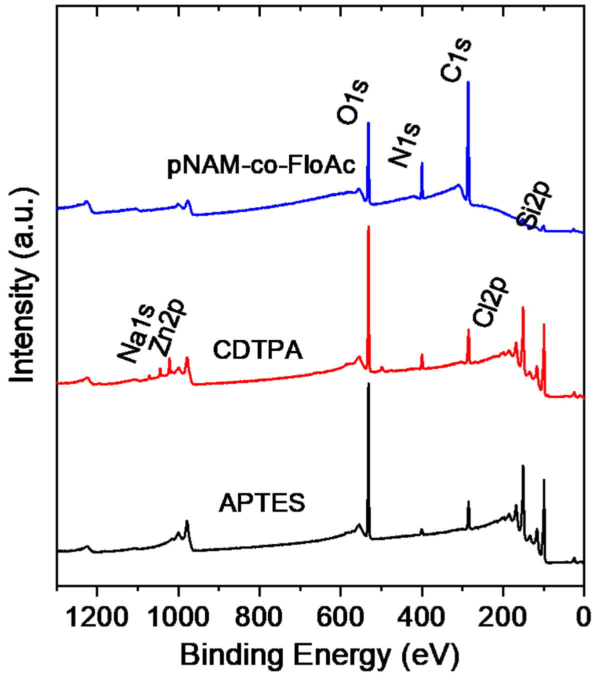


**Figure S7.** XPS survey spectra of APTES, CDTPA and pNAM-*co*-FloAc 60kDa

**Table S2.** Apparent surface chemical composition of APTES, CDTPA and pNAM60kDa determined from XPS survey spectra.

| Sample | Surface chemical composition (at.%) | | | | | | |
|---|---|---|---|---|---|---|---|
| | **C1s** | **O1s** | **N1s** | **Si2p** | **Cl2p** | **Zn2p** | **Na1s** |
| Si wafer (reference) | 4.3 | 37.1 | - | 58.7 | | | |
| APTES | 15.8 ± 0.4 | 32.7 ± 0.3 | 3.5 ± 0.3 | 47.4 ± 0.6 | 0.6 ± 0.2 | | |
| CDTPA | 20.6 ± 1.7 | 30.7 ± 0.7 | 5.2 ± 1.0 | 42.5 ± 3.0 | 0.3 ± 0.3 | 0.5 ± 0.4 | 0.3 ± 0.2 |
| pNAM60kDa | 65.8 ± 2.7 | 19.7 ± 0.7 | 9.8 ± 0.2 | 4.7 ± 1.7 | | | |

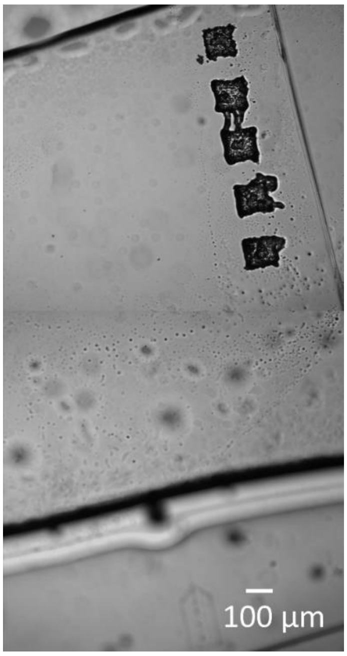


**Figure S8.** Transmission CLSM image of pNAM-*co*-NABAAm prepared via the 2PRAFT polymerization with the 4 M formulation containing NAM and NBAAm at scanning speed 100 mm.s$^{-1}$ and power 80 mW. Visible dark squares indicate thermal damage caused by increased layer spacing.

## 6. Two-coloured image of end-group cleaved and labeled pNAM-pFloAc brushes on glass surface

After the surface modification and 2PP printing according to reported procedure, the glass cover slip substrates were further end-group modified via aminolysis by immersion in a solution of n-propylamine (nPr-$NH_2$; 1 mL; 12.2 mmol) and tris(2-carboxyethyl)phosphine hydrochloric acid salt (TCEPxHCl; 20 mg; 0.07 mmol) in dry DCM (40 mL) for 12 h. The substrates were cleaned with DCM and dried under argon stream. Subsequently, the substrate was incubated for 15 min in a diluted solution of Alexa Fluor 647 $C_2$ Maleimide (0.01 mM) and TCEPxHCl (0.5 mg/mL) in PBS buffer and cleaned with fresh PBS buffer and argon stream before analysis via CLSM imaging (see scheme in Figure S9).

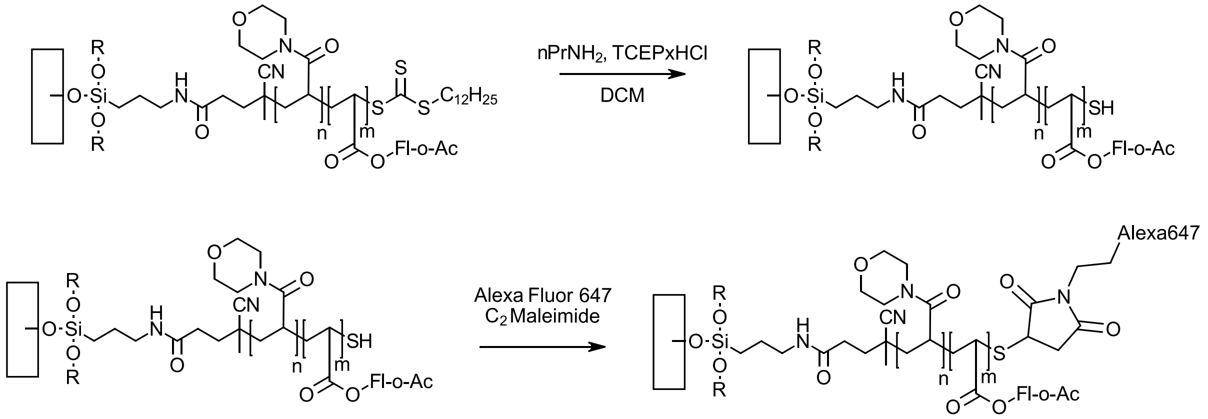


**Figure S9.** Scheme of end-group cleavage and end- group labeling of modified surface to prove livingness of chain ends.

The CLSM image (Figure S10) confirm the successful end-group labeling due to strong overall fluorescence in the red channel as well as the preservation of the polymer brush structures in the green channel. Two areas of 15 x 15 pixel corresponding to 150 µm$^2$ were chosen, one directly on the letter B, one further away from the printed structure. The intensity differences in the green channel are obviously high since the blank signal is only noise, but the insignificant difference in the red channel of only 1 % (see Table S3) confirms the presence of thiol groups on the grafted polymer chains, otherwise the intensity difference must be higher. Based on the performed experiments, the livingness of the chain ends was confirmed.

.

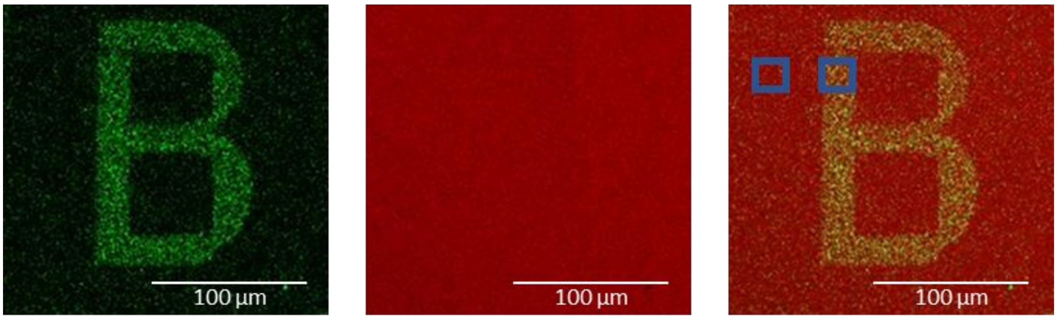


**Figure S10.** Printed letter B at 100 mm/s, 80 mW, 0.2 µm layer spacing - CLSM images after end-group labeling with Alexa Fluor 647 $C_2$ Maleimide: Green channel for Fl-o-Ac (left), Red channel for Alexa Fluor 647 (middle) and overlay image with areas for fluorescence intensity comparison (right)

**Table S3.** Relative fluorescence intensity of defined areas from the CLSM measurement

| | Area [$\mu m^2$] | Rel. Intensity [%] |
|---|---|---|
| **Red blank** | 150 | 99 |
| **Red letter B** | 150 | 100 |
| | | |
| **Green blank** | 150 | 37 |
| **Green letter B** | 150 | 100 |

## 7. References


[1] E. Mutlutürk, T. Caykara *Turk J Chem* **2023,** *47* (1), 185-195. DOI 10.55730/1300-0527.3528.

[2] S. F. Helfert. Synthesis and characterization of polymer-linker systems for T-cell activatioc. Diploma thesis, Technische Universität Wien, **2017**.

[3] W. J. Brittain, S. Minko *Journal of Polymer Science Part A: Polymer Chemistry* **2007,** *45* (16), 3505-3512. DOI https://doi.org/10.1002/pola.22180.

[4] O. G. Andriotis, W. Manuyakorn, J. Zekonyte, O. L. Katsamenis, S. Fabri, P. H. Howarth, D. E. Davies, P. J. Thurner *J Mech Behav Biomed Mater* **2014,** *39*, 9-26. DOI 10.1016/j.jmbbm.2014.06.015.
